# Authors’ response: ‘Implementation of facemask sampling for the detection of infectious individuals with SARS-CoV-2 in high stakes clinical examinations – a feasibility study’

**DOI:** 10.1016/j.fhj.2025.100227

**Published:** 2025-02-15

**Authors:** Daniel Pan, Manish Pareek, Michael R. Barer

**Affiliations:** a Department of Respiratory Sciences, University of Leicester, United Kingdom; b Department of Infectious Diseases and HIV Medicine, University Hospitals of Leicester NHS Trust, United Kingdom; c NIHR Leicester Biomedical Research Centre, United Kingdom; d Development Centre for Population Health Sciences, University of Leicester, United Kingdom; e Department of Clinical Microbiology, University Hospitals of Leicester NHS Trust, United Kingdom

We thank Daungsupawong and Wiwanitkit for their interest in our manuscript. Our primary aim in this piece of work was to assess the acceptability of facemask sampling (FMS) within clinical settings.^1^ Questions raised by Daungsupawong and Wiwanitkit relating to sensitivity and specificity, as well as differences in viral load detected in FMS and upper respiratory tract sampling (URTS), were not the focus of this work. We note some relevant points from our other publications^2^, ^3^, ^4^ below:

• Viral load kinetics of FMS and URTS are different during infection.
• URTS is positive for longer periods of time following infection compared to FMS (ie URTS appears to be more sensitive than FMS for *diagnosis* of SARS-COV-2).
• FMS associates with household transmission of SARS-COV-2 better than URTS following natural infection (ie FMS appears to be better than URTS at detecting those who are *infectious* with SARS-CoV-2).

Therefore, the desire of most study participants for confirmatory testing using URTS of a positive FMS result in this manuscript should not affect the use of FMS, since we foresee the two sampling modalities to have different clinical applications. A positive URTS confirms the diagnosis of SARS-CoV-2, while FMS would assess how infectious the same individual is to others. A non-infectious individual who has recovered from acute infection (ie URTS positive, but no longer FMS positive for instance) could then return to work sooner than requiring a negative URTS result. Furthermore, if FMS replaced standard facemasks in hospitals for healthcare workers, they may be able to detect those who test positive for SARS-CoV-2 prior to symptom onset, and possibly even before a positive URTS sample. An isolation policy developed based on timely FMS results could have significant impact on nosocomial transmission of respiratory viruses.

In summary, the biological questions raised by Daungsupawong and Wiwanitkit were not the focus of our paper and are discussed elsewhere. The current paper presents findings relating to acceptability of FMS for detecting infectious individuals within nosocomial settings, which, in combination with other work, will be crucial for designing better, more precise interventions that can reduce respiratory virus transmission within hospitals.

## Funding

DP is supported by an NIHR Doctoral Research Fellowship (NIHR302338). The views expressed are those of the author(s) and not necessarily those of the NIHR or the Department of Health and Social Care.

## CRediT authorship contribution statement

**Daniel Pan:** Conceptualization, Writing – original draft, Writing – review & editing. **Manish Pareek:** Supervision, Writing – review & editing. **Michael R. Barer:** Supervision, Writing – review & editing.

## Declaration of competing interest

The authors declare that they have no known competing financial interests or personal relationships that could have appeared to influence the work reported in this paper.
